# Barriers to modern contraceptive methods uptake among young women in Kenya: a qualitative study

**DOI:** 10.1186/s12889-015-1483-1

**Published:** 2015-02-10

**Authors:** Rhoune Ochako, Mwende Mbondo, Stephen Aloo, Susan Kaimenyi, Rachel Thompson, Marleen Temmerman, Megan Kays

**Affiliations:** Population Services Kenya (PS Kenya), P.O. Box 22591, 00400 Nairobi, Kenya; Population Services International, P.O. Box 14355, 00800 Nairobi, Kenya; International Centre for Reproductive Health, Ghent University, Ghent, Belgium

**Keywords:** Family planning, Kenya, Young women, Contraceptives, Myths and misconceptions, Behaviour change communication

## Abstract

**Background:**

Young women in Kenya experience a higher risk of mistimed and unwanted pregnancy compared to older women. However, contraceptive use among youth remains low. Known barriers to uptake include side effects, access to commodities and partner approval.

**Methods:**

To inform a youth focussed behaviour change communication campaign, Population Services Kenya developed a qualitative study to better understand these barriers among young women. The study was carried out in Nyanza, Coast, and Central regions. Within these regions, urban or peri-urban districts were purposively selected based on having contraceptive prevalence rate close to the regional average and having a population with low socioeconomic profiles. In depth interviews were conducted with a sample of sexually active women aged 15–24, both users and non-users, that were drawn from randomly selected households.

**Results:**

All the respondents in the study were familiar with modern methods of contraception and most could describe their general mechanisms of action. Condoms were not considered as contraception by many users. Contraception was also associated with promiscuity and straying. Fear of side effects and adverse reactions were a major barrier to use. The biggest fear was that a particular method would cause infertility. Many fears were based on myths and misconceptions. Young women learn about both true side effects and myths from their social networks.

**Conclusion:**

Findings from this research confirm that awareness and knowledge of contraception do not necessarily translate to use. The main barriers to modern contraceptive uptake among young women are myths and misconceptions. The findings stress the influence of social network approval on the use of family planning, beyond the individual’s beliefs. In such settings, family planning programming should engage with the wider community through mass and peer campaign strategies. As an outcome from this study, Population Services Kenya developed a mass media campaign to address key myths and misconceptions among youth.

## Background

Several decades after the introduction of modern family planning methods, Kenya’s population is still growing and is projected to exceed 60 million by 2025 [1]. Although fertility declined between 1978 to 1997, it has levelled off in recent years [2]. This stall is attributed to a number of factors including reduced availability of modern contraceptive methods, diversion of resources to HIV/AIDS, and inadequate support for family planning programmes [2,3]. According to the Kenya Demographic Health Survey (DHS) 2008–09, total fertility rate (TFR) was 4.6, while 42% of married women reported their current pregnancies as unintended [4]. Contraceptive prevalence was found to be 46%; a result that did not meet the 2010 target of 62% set by the Kenya National Population Policy for Sustainable Development [1,3].

In the last decade, youth fertility has declined by 7% but the contribution to overall fertility (TFR) has increased from 32% in the late 1970s to 37% in 2008 [1]. The proportion of teenagers who have started childbearing increases from 2% at age 15 to 36% by age 19 [4]. According to the Kenya DHS 2008–09, 12% of women aged 20–49 had sex before age 15, and about half had their first sex by their 18th birthday [4]. Research by the Centre for Study of Adolescence found that four in ten Kenyan girls had sex before the age of 19, many of them as early as 12 [5]. Recent statistics from the Ministry of Planning indicate that 97% of males and 85% of females aged 15–19 years are not married [1]. This suggests that age at first marriage cannot be used as a proxy for age at first sex [4] and many young people are having sex before marriage.

Young women in Kenya experience a higher risk of mistimed and unwanted pregnancy compared to older women. While the total mistimed (26%) and unwanted (17%) pregnancies among all women (15–49 years) remains high, young women (15–24 years) experience even higher mistimed (32% vs. 30%) and unwanted (15% vs. 10%) pregnancies compared to women in other age groups [4]. Every year, about 13,000 Kenyan girls drop out of school due to accidental pregnancy [5] and 103 out of every 1000 births in Kenya are delivered to girls aged 15–19 [1]. Accidental pregnancy is a leading cause of abortion [6]. However, contraceptive use remains low among youth; 73% of currently sexually active single women aged 15–19 report not using any contraception method [4].

Across all age groups, perceived and actual side effects of contraceptive methods emerged as a primary barrier to use. Kenya’s DHS (married women only) found that non-users who did not intend to use contraception in the future most commonly cited fear of side effects and health concerns [4]. Side effects are also the most common reason for method discontinuation [4,7].

Even when awareness is high, poor knowledge of contraceptive methods and their side effects has been associated with poor uptake [8]. This finding may be related to the myths and misconceptions that many women hold about potential side effects and negative outcomes [9-11]. Myths are heard about from peers and partners, whose influence on contraceptive demand and uptake is well documented in Kenya [8,11-13].

Another key barrier is lack of physical and financial access to family planning commodities. Studies have shown that health facilities offering family planning are not equitably distributed throughout the country [3]. Women complain of frequent stock-outs and the associated costs of lost wages, transport and other financial challenges [8,11,14]. Studies have shown that, among youth, lower socioeconomic status has been associated with less condom use [15,16].

Shame is also a significant factor preventing use of family planning (specifically condoms), particularly for unmarried youth. Young people perceive women who carry condoms as promiscuous [17], and that asking a partner to use condoms would reveal them as sexually wayward or untrustworthy [6]. Young people also noted that while married people may freely ask for family planning, they are inhibited because of the shame associated with procuring contraceptives [6,18].

At service level, many providers and available health information indicate that family planning are only for those who are “mothers”, and are not suitable for those who have not yet had a child [14]. At the policy level, a recent commentary in the Lancet advocates for the replacement of the term “family planning” with “contraception”; a more neutral term that applies to users, with or without families [19].

Despite the high proportion of unmarried sexually active youth in Kenya, the majority of research on barriers to family planning has been conducted among married women; research among youth is limited to condoms only [7,19,20]. Communications target married women and highlight the need to limit family size. Behaviour change communications do not respond to youth, whose needs are mainly to delay childbearing, and there is a gap in active and consistent national communication to create awareness and demand for family planning among 15–24 year olds.

In response, Population Services Kenya, in collaboration with the Division of Reproductive Health (now Reproductive and Maternal Health Services Unit), launched a communication campaign to demystify contraceptives among youth. To inform this new campaign, PS Kenya developed a qualitative study to better understand the drivers and barriers to contraceptive uptake among young women.

## Methods

This study employed a qualitative approach using in-depth interviews with key members of the target population. In-depth interviews were selected given the sensitivity of discussing sexual activity among young people; one-on-one sessions offered participants the chance to speak more freely and offered the interviewer the opportunity to probe about barriers and drivers to contraceptive use.

### Setting

PS Kenya’s programmatic focus is on young women aged 15–24 in urban and peri-urban areas who come from lower socioeconomic classes, given the high unmet need in this segment of the population [4]. The Living Standards Measure (LSM) approach that assesses social economic status (SES) through ownership of various assets by the respondent was used. LSM, a widely used marketing research tool to segment population, is based on scores from ownership of various assets. We subdivided our population into 4 SES groups from the highest to lowest; AB, C1; C2 and DE. We screened and recruited respondents from social classes C1, C2 and DE where PS Kenya programmes intervenes. Other parameters (female sex, age 15–24, and urban/peri-urban residence) formed the eligibility criteria for inclusion in the study, along with sexual activity in the last twelve months.

Respondents for the study were drawn from three regions of Kenya: Nyanza, Coast, and Central. These three regions were chosen based on contraceptive prevalence rates (CPR) in the regions as compared to the national average (39%); Central has a higher CPR (67%), Nyanza near average (37%) and Coast, the lowest CPR at 34% [4].

### Sampling

Respondents were drawn from purposively selected districts within the three regions. Districts were selected based on having CPRs similar to the regional average, being primarily urban or peri-urban, and having a population with low socioeconomic profiles. Within each district, a list of locations and sub-locations was generated, and one sub-location in each district was randomly selected and further smaller administrative units, villages/estates, were randomly selected where respondents were screened and recruited if they met the eligibility criteria.

Field-based recruiters assisted with identifying the women within the villages/estates. A landmark within the villages/estates was randomly selected, and field recruiters visited households in a predetermined direction. Each fifth household was approached to see if they had an eligible respondent. No more than one respondent per household was recruited, and if there was more than one eligible woman the youngest was taken. This process was repeated until a pre-set quota of women was reached. The recruiters then set a date for the interviewer to return to conduct the interview. The refusal rate for this survey was negligible and comparable to that of larger household surveys conducted in Kenya such as the Kenya Demographic and Health Survey and the Kenya AIDS Indicator Survey that record response rates of over 96%.

### Study population

As shown in Table 1 below, 34 young women interviewed for this study were from three sampled locations of Kisumu, Thika and Mombasa. These women were from the lowest socioeconomic class who are the target group for PS Kenya interventions. While the study intended to recruit a higher proportion of non-users than users, in fact more users (58.8%) were recruited because some women who used condoms did not consider them as a method of contraception. The majority of the women (61.8%) were aged 20–24, with the rest 16–19 years. Most women had secondary education (44.1%) and were unemployed (44.1%); 29.4% were currently students. The majority of the women had no child (52.9%) and were single (55.9%).

**Table 1:** **Characteristics of the study population**

**Characteristics**	**Percent**	**N**
**Age**		
16-19 years	38.2	13
20-24 years	61.8	21
**Education**		
Primary	35.3	12
Secondary	44.1	15
College	20.6	7
**Marital status**		
Single	55.9	19
Married	44.1	15
**Contraceptive use**		
User	58.8	20
Non user	41.2	14
**Number of children**		
None	52.9	18
1 child	32.4	11
2-4 children	14.7	5
**Occupation**		
Student	29.4	10
Unemployed	44.1	15
Casual job	8.8	3
Business	17.7	6
**Study location**		
Kisumu	38.2	13
Mombasa	38.2	13
Thika	23.6	8

### Data collection

The data was collected by a team of experienced and trained qualitative interviewers in April 2012. The interviews took place in a location selected by the respondent, preferably in their homes. The interviews were based on a discussion guide that covered the main topic explored in this paper, beliefs to change, which are narratives from the participants through which they expressed costs and disadvantages of modern contraceptive use. Other topics explored during discussion with the participants were: archetype (demographic facts, values, aspirations, worries and fears); beliefs to reinforce (benefits of contraceptive use); strategies to behave (statements with intent to behave, an obstacle and how to overcome the obstacle); acquisition stories (how they buy or receive modern contraceptives); category experience (past and present experiences and other competing behaviours); knowledge and sophistication on various methods; openings (preferred media channels for communication); and brand associations (emotional attachments to specific contraceptive brands). The interview guide was drafted in English and translated in Kiswahili. Interviews were conducted in English and Kiswahili by trained and highly experienced qualitative Research Assistants. Interviews were audio recorded.

### Data analysis

Interviews were transcribed and translated into English where necessary. The data was coded using a set of pre-set codes based on the discussion guide as well as emergent themes. The thematic coding framework was then applied to assess all interview transcripts. The analysis looked for patterns and associations on the emerging themes, focusing on the drivers and barriers to modern family planning uptake. Quotations from the study participants have been used to characterize emerging issues and themes. Non-identifying information of the study participants has been used in this paper. RATS guideline for reporting qualitative studies were adhered at all points during the study.

### Ethical considerations

Permission to conduct this research was obtained from the Kenya National Council of Science and Technology (now National Commission for Science, Technology and Innovation) while ethical approval was obtained from the Population Services International Research and Ethics Board. As per ethical regulations that govern research involving participants who are considered as minors, consent was sought from parents/guardians of all participants aged below 18 years, the age of consent in Kenya, before their participation in the study. Verbal consent was provided by the study participants, and no identifying information was connected with the interviews or retained following the completion of the analysis.

## Results

### Knowledge and experience of family planning

All the respondents in the study were familiar with modern methods of contraception. Among current and former users, most personal experiences were with the IUD (the coil), condoms, pills, injectables and implants. Most women were able to name several forms of contraception and to describe, in general, their mechanisms of action. However, knowledge of dual protection varied:

Many prefer the condom since it prevents in two ways, she (person using) won’t get pregnant, she also believes she won’t contract any diseases [User, Mombasa]

To prevent those, I mean early pregnancies, young girls, to the teenage they should use those coils, the contraceptives to prevent early pregnancy, STI and AIDS [Non-user, Kisumu]

In other cases, respondents were familiar with different methods, but not with how they were appropriately used.

The woman inserts it (pill in the vagina) so that she doesn’t get pregnant [Non-user, Kisumu]

Those things are harmful, I saw once, one have to be cut open somewhere here and they insert the coil then they stitch the area, I don’t know those are the coils and it last for I don’t know twenty years [Non-user, Kisumu]

In terms of their personal experience with family planning, it is interesting to note that not all participants correctly identified themselves as ‘users’ or ‘non-users’: those who used condoms described themselves as ’non-users’. This finding is based on the perception that condoms are not reliable for pregnancy prevention due to the potential for the method’s failure. The quote below illustrates this view:

I always thought that condoms were used to prevent diseases like HIV but not pregnancies because I was using the condoms but I still got pregnant. The day it burst is the day I conceived. So I would not say that a condom is a family planning method [Non-user, Mombasa]

### Barriers to family planning use

#### Myths about contraceptives

Among the respondents, fear and concerns about family planning were a major barrier to use. Many of their fears were based on myths and misconceptions. The largest concern cited by participants was fear that a particular method would render them infertile; in many cases, this prevented them from using contraception.

I think the pills are not good and even my mum has warned me severally not to use pills because there will come a time when I might need to have a children and I might not be able to get one in future. You don't get pregnant (IF YOU USE PILLS) [Non-user, Mombasa]

If they put that (implants) on you when you remove it (implants) you cannot give birth again [User, Kisumu]

Some say that you, you might never give birth if you are used to taking the injections [Non-user, Kisumu]

While infertility was cited as a possible consequence of most methods, it was most strongly expressed around injectables. As a result, many young women believed that injectables were only recommended for women who already had children.

There are some who love the injection but they advise the ladies who are here that they should only go for the injection only if they have ever given birth before, that it’s not good to go if they’ve never given birth, they should go for the injection after [Non-user, Kisumu]

Participants also believed that it was possible that modern contraception methods could cause temporary infertility or reduce one’s childbearing capacity, limiting the number of children they were able to conceive in their lifetime.

I have little faith in pills…it (pills) affects me in that I cannot be able to give birth to many children [User, Mombasa]

She (mum) said it (pills) can cause infertility…can take long before conceiving [Non-user, Kisumu]

Another cited barrier to family planning use is the association of modern contraceptives with birth defects or abnormalities.

I think people like the injection more…Pills are very bad and I don’t like anything to do with them…if you take the pill for so long, you may give birth to a paralyzed child… [User, Mombasa]

You hear that someone was pregnant and they got pregnant while using the pills and then you start worrying if you will give birth to a normal child or you will give a child without hands [Non-user, Mombasa]

A number of participants expressed concerns about contraceptives as a foreign object that could disrupt the natural processes of the body and create harm. Failure to menstruate regularly, a common side effect from using certain contraceptive methods, was interpreted as causing the body to retain ‘dirty blood’ and leading to stomach aches.

Some fear that they will not receive their period when they use them (referring to contraceptives) and that blood is dirty and should come out [User, Kisumu]

Condoms were associated with discomfort and irritation from the lubricant, which they feared may cause an infection. Methods such as the coil or implant that were inserted were seen as having the potential to harm one’s internal organs.

A large number of respondents, especially those from Kisumu, also reported having heard or believing that the use of certain contraceptive methods, especially pills, led to cancer.

Many of them (community women) say that the pills are not good because they can accumulate under the abdomen and cause something like cancer; so many people don’t like those pills [Non-user, Kisumu]

#### Fear of side effects

In addition to the myths described above, many also mentioned real side effects as a barrier to use. The most common side effects expressed by the respondents were weight changes, bleeding, and lack of sexual desire. Headaches and blood pressure issues were also cited by a few.

All of the methods were associated with potential changes in weight, with some methods associated with weight gain (notably the injectable) and others with weight loss.

Injectable, I feel they are not good as they have side effects; they change your physical appearance, very fat, very many people get very fat [User, Mombasa]

I don’t know if its maybe I used them (pills) for a long time the first time and that’s why they (pills) are affecting me? They (pills) make me loose appetite and I start getting thin because I don’t eat [User, Mombasa]

Heavy bleeding was also associated with modern family planning methods.

The periods are so heavy till they feel like they are not menstruating, it’s like they are bleeding pure blood. [Non-user, Mombasa]

And others I heard that they receive their periods like the entire month when they are using these family planning methods. Like Norplant there is a friend who had periods throughout the month until she had it removed [Non-user, Mombasa]

Respondents also reported hearing that modern family planning reduces sexual desire.

All these family planning methods interfere with our feelings (libido) be it a coil, be it a tablet, (pills) be it what, I heard that it (modern family planning) reduces feelings [User, Mombasa]

Injectable I heard they make a woman “to be cold”. They lead to lack of sexual arousal just like the coil. There are some who have side effects by the way you “don’t have any feelings (sexual urge) [Non-user, Mombasa]

Sometimes they complain because of the way they (pills) make you feel tired, bring mood swings and sometimes you have a low libido. This will make him complain because he will start accusing you that you are being unfaithful to him [User, Mombasa]

#### Association with promiscuity and straying

There were notions that the use of modern contraceptive methods encouraged young women to become sexually promiscuous. Both users and non-users expressed the belief that the partners of young women who use contraceptives felt that they encouraged the women to be unfaithful. For instance, a non-user from Mombasa stated:

And so they (men) say that only promiscuous women use the pills and that is why they are against those pills [Non-user, Mombasa]

However, this seemed to be a belief held by the male partners and not the women themselves. Another respondent from Mombasa, this time a user, also stated that:

Sometimes they (men) accuse you of being unfaithful in the relationship [User, Mombasa]

The issue of modern contraceptives being associated with lack of trust was reported to have been discussed during youth meetings in the community, as mentioned by one of the respondents who said:

We have discussions at some youth meetings we go for, they say that using the condoms means that you don’t trust your girlfriend [Non-user, Kisumu]

Another respondent remarked that:

With the pills they (men) are very… much against it and they feel that with the pills their women can have extra marital affairs knowing that they will not get pregnant [Non-user, Mombasa]

Some young women thought that condoms were not a suitable method of contraception for couples in relationships because of their association with straying.

They (community members) don’t use condoms because they are married and you cannot use them with your husband when you are married. Maybe if you are having an affair, I don’t like condoms because, I have a husband that I live with so I don’t see why I should use condoms and I am not straying. [Non-user, Mombasa]

### The role of others in influencing contraceptive use

As demonstrated by the repeated use of the third person pronouns (‘he’, ‘she’, ‘they’, ‘others’) in almost all of the above quotes, young women learn about both true side effects and myths from others in their community - peers, family or partners. Often these women received inaccurate information and were directly counselled by others not to engage with or use family planning methods.

They don’t like it (modern FP) at all, they tell me not to use pills, don’t use family planning, it will affect your fertility…when they hear that a girl has been taken to get an operation maybe she has an appendix…they will say that it has been caused by family planning [User, Mombasa]

I heard of a woman who got pregnant while using the coil. And she had some difficulties while giving birth, she had to go to the hospital and undergo an operation [Non-user, Mombasa]

If the advice I get from the community is good then there is no need to go to hospital, in my community we don’t use pills or the injection but we do it on our own and we are able to plan properly [Non-user, Mombasa]

I used to hear people say that they’re (modern FP methods) bad especially when I was a girl they’d say it would harm my body, like being unable to conceive [User, Kisumu]

Women also shared concerns raised by their spouses or partners on use of contraceptives, which echoed their own concerns about outcomes and side effects. Male partners raised concerns about family planning diminishing sexual urges, resulting in birth defects, or discomfort during sex.

There are some men who don’t want their women to use the planning methods, some men say that the medicines will result in messing up their reproductive organs [User, Thika]

They (men) say that when you get the injection you are no longer aroused, Some (men) say when they use the condom it affects that thing (penis), When he is through he feels a lot of pain the next day [User, Kisumu]

There was also a suggestion that men prevent women from using family planning to increase the number of their progeny.

Men do not like because they (men) want their wives to give birth to as many children as possible as they can [Non-user, Kisumu]

## Discussion

Most respondents are aware of contraceptives and, generally, knowledge of modern contraceptive methods was high. The majority of respondents knew at least one modern method of contraception. This is in line with findings from the Kenya DHS that demonstrated knowledge of contraceptives is almost universal [4]. Yet despite this high level of knowledge, use - particularly among young women - remains low. This finding that knowledge of contraception is not necessarily correlated with use has been shown in the research of others [9,20].

Knowledge of use and potential side effects varied between respondents, and between methods. For example, although the majority of respondents correctly understand that condoms offered dual protection, some felt that condoms were not reliable for pregnancy prevention. Furthermore, some participants who used condoms did not describe themselves as ‘users’ of family planning. This suggests that the condom is not classified as a method of contraception.

Many women report hearing about health related problems from the use of contraceptives, including: total or temporary infertility, birth defects and abnormalities, disruption of their normal body processes or inability to menstruate regularly. Similar findings of health related concerns in Kenya are reported in a study on discontinuation of injectables [14]. The Kenya DHS reports health related concerns as the second most common reason for non-use or contraceptive discontinuation [4].

Side effects were mentioned by a majority of the young women as one of their greatest fears. The main side effects mentioned were weight gain, lack of sexual desire, headaches and blood pressure. These results are supported by the Kenya DHS that found, overall, 36% of women report discontinuation within the first 12 months of using a method due to side effects; and 16% of married women not currently using were not doing so due to fear of side effects (Kenya National Bureau of Statistics (KNBS) and ICF Macro 2009). Disruption of menstrual cycle was the most common reported reason for discontinuation of hormonal methods among women in Nyando district, Kenya [14]. A study in Tanzania also found fear of side effects as the main reason for discontinuation and non-use of family planning [21].

While previous studies have focused on user and method related reasons for discontinuation or non-use, little focus has been placed on the influence of others [14]. In our research, health providers and other educational sources were rarely mentioned as the source of information on contraception. Instead, peers and other community members acted as the main sources, and their perceptions also heavily influenced the decision to use or not. However, as the quotes show, these sources often propagated myths (infertility, birth defects) and exaggerated rare side effects (uncontrollable bleeding, enormous weight gain/loss). These findings are in line with other studies, for example, the influence of others’ perceptions - propagated through hearsay - was also found in a study among youth in Kisumu [9]. Meanwhile, other studies report a similar over exaggeration of side effects as a result of myths and misconceptions held by a community [21,22].

Our findings support evidence that women do not make decisions to use contraceptives in isolation, but in consultation with others in their social networks [10,12,13,23]. Both information and misinformation are spread through social networks. In this way, networks provide an opportunity to encourage or discourage use; a way of sharing potentially positive information on contraceptive technologies but also a channel for rumours, which may negatively influence use [23,24]. The spread of myths by social networks in the community is demonstrated by a longitudinal study in Nyanza, Kenya [23,25]. Myths are learned from childhood: a project collecting anonymous questions from children in Kenya demonstrated several misconceptions about the efficacy of condoms [26]. An interesting finding in Kenya, which our quotes also suggest, is that men and women were influenced more by their perception of their social network’s approval of family planning than by their own approval [23].

Within the social network, our findings point to the importance of partner views in determining use or non-use of modern contraceptives. In a study in Kenya, partner’s influence was found to be a key barrier, based on the husband’s desire to exert influence on childbearing and unfounded concerns about family planning methods [13]. Partners were reported not to accept the use of some methods because they associated them with poor health, infertility, birth defects, infidelity and promiscuity [14]. Contraceptives were also seen by partners to reduce a woman’s libido, thereby interfering with marriage, and sometimes resulting in less pleasure during sex [13]. A study in Nyanza, Kenya, further confirms that women do not always have control on the start and discontinuation of a contraceptive; it is their husbands or other influential people in their community or household who make the decision [10]. Much of this opposition, they report, was due to low levels of knowledge regarding side effects and cultural beliefs around sex and fertility [10].

### Programmatic implications

Demographers and health planners have typically based programmes on ’individualistic rational -actor models’ [25]. However, as this and other research suggests [10,12,13,23], social networks are key in perpetuating myths that act as barriers to uptake. These findings stress the influence of social network approval on the use of family planning, above the individual’s beliefs. In such settings, family planning programming should utilize innovative strategies to override myths and rumours, for example, intensive couple and peer counselling [12]. This research also highlights the importance of engaging with the wider community (mass strategies and peer strategies) in programming, as well as the importance of understanding the way information is spread informally in Kenyan society. Especially among youth, social media offers a new opportunity to spread information rapidly and effectively, in order to remove barriers and drive demand for contraception.

As an outcome from this study, PS Kenya developed a campaign called the ‘C-word’. This national campaign includes messages to address key myths and misconceptions identified, specifically: Contraceptives cause weight gain; Contraceptives can cause infertility; Condoms are not effective in preventing pregnancy; Contraception is women's business; and Contraceptives can cause cancer. These are currently being exposed to the target audience through radio, TV, online and social media. Additionally, interpersonal communication activities on the ground seek to challenge beliefs that regular contraceptives are for older women only, contraceptives are not safe, will cause infertility, as well as other method specific issues exposed by this study. The aim of all these activities is to address misinformation and increase modern contraceptive uptake among young Kenyans.

## Conclusions

Findings from this research confirm that awareness and knowledge of contraception do not necessarily translate to use. The main barriers to modern contraceptive uptake among young women in Kenya are myths and misconceptions, with both users and non-users exhibiting lack of factual information on the different contraceptive methods. Social networks influence contraceptive use by exaggerating side effects and spreading myths.

The data from this study highlights the social nature of beliefs and behaviours around family planning. The decision to use or not is primarily influenced by others from within the social network, whose views and perceptions are often more important than an individual’s own. Therefore, family planning campaigns should look beyond the individual - to social networks - in order to drive demand and remove barriers.

The influence of social networks on health behaviours is relatively under-researched. Exploring the mechanisms by which social networks influence decision making is thus suggested as an area for future research. To complement this analysis, anthropological research to examine the cultural norms that underlie social networks is also recommended, to help develop programmes that can target the cultural barriers to family planning in Kenya.

Partner influence also remains key and so it is important that myths and unfounded concerns raised by male partners are addressed, for example, by designing male friendly interventions. We recommend that the Ministry of health takes up these findings by coming up with policies on comprehensive sexuality education that will also include facts about contraception. This study focussed on young women only. Further research with men, whose influence on contraception demand and uptake has been well noted is, therefore, recommended to help inform new programming.
